# Galectin-3: A Friend but Not a Foe during *Trypanosoma cruzi* Experimental Infection

**DOI:** 10.3389/fcimb.2017.00463

**Published:** 2017-11-03

**Authors:** Aline A. da Silva, Thaise L. Teixeira, Samuel C. Teixeira, Fabrício C. Machado, Marlus A. dos Santos, Tatiana C. Tomiosso, Paula C. B. Tavares, Rebecca T. e Silva Brígido, Flávia Alves Martins, Nadjania S. de Lira Silva, Cassiano C. Rodrigues, Maria C. Roque-Barreira, Renato A. Mortara, Daiana S. Lopes, Veridiana de Melo Rodrigues Ávila, Claudio V. da Silva

**Affiliations:** ^1^Departamento de Imunologia, Instituto de Ciências Biomédicas, Universidade Federal de Uberlândia, Uberlândia, Brazil; ^2^Departamento de Microbiologia, Imunologia e Parasitologia, Escola Paulista de Medicina, Universidade Federal de São Paulo, São Paulo, Brazil; ^3^Faculdade de Medicina de Ribeirão Preto, Universidade de São Paulo, Ribeirão Preto, Brazil; ^4^Laboratório de Bioquímica e Toxinas Animais, Instituto de Genética e Bioquímica, Universidade Federal de Uberlândia, Uberlândia, Brazil

**Keywords:** galectin-3, *Trypanosoma cruzi*, heart, fibrosis, leukocytes

## Abstract

*Trypanosoma cruzi* interacts with host cells, including cardiomyocytes, and induces the production of cytokines, chemokines, metalloproteinases, and glycan-binding proteins. Among the glycan-binding proteins is Galectin-3 (Gal-3), which is upregulated after *T. cruzi* infection. Gal-3 is a member of the lectin family with affinity for β-galactose containing molecules; it can be found in both the nucleus and the cytoplasm and can be either membrane-associated or secreted. This lectin is involved in several immunoregulatory and parasite infection process. Here, we explored the consequences of Gal-3 deficiency during acute and chronic *T. cruzi* experimental infection. Our results demonstrated that lack of Gal-3 enhanced *in vitro* replication of intracellular parasites, increased *in vivo* systemic parasitaemia, and reduced leukocyte recruitment. Moreover, we observed decreased secretion of pro-inflammatory cytokines in spleen and heart of infected Gal-3 knockout mice. Lack of Gal-3 also led to elevated mast cell recruitment and fibrosis of heart tissue. In conclusion, galectin-3 expression plays a pivotal role in controlling *T. cruzi* infection, preventing heart damage and fibrosis.

## Introduction

*Trypanosoma cruzi* is an obligate intracellular parasite able to infect and replicate within different mammalian cells with tropism for cardiomyocytes and smooth muscle (Zhang and Tarleton, 1999; Epting et al., 2010). This parasite is the causative agent of Chagas' disease and according to the World Health Organization, there are about 6–7 million infected people worldwide (World Health Organization, 2016). Chronic chagasic cardiomyopathy (CCC) is the most severe form of Chagas' disease and is marked by heart failure, ventricular arrhythmias, heart blocks, thromboembolic events, and sudden death (Moncayo and Silveira, 2009). The pathology and worst prognosis has been attributed to parasite persistence and immune-mediated mechanisms, leading to continuous myocardial damage, and interstitial fibrosis (Rassi et al., 2009).

Host cells provide a favorable environment for parasite perpetuation and host immune response evasion (Sibley, 2004). After entering host cells, *T. cruzi* is transiently retained within a parasitophorous vacuole that rapidly fuses with host cell lysosomes (Tardieux et al., 1992; Woolsey et al., 2003). The parasites escape from the phagolysosome to the host cell cytoplasm where they replicate until new trypomastigotes lyse the cells and are released into the bloodstream, from where they can invade any nucleated cell to begin a new reproductive cycle (Nogueira and Cohn, 1976; Ley et al., 1990; Cardoso et al., 2015). Recently, we have reported that internalized trypomastigotes that have escaped from the parasitophorous vacuole transiently accumulate galectin-3 that acts as a marker of *T. cruzi* phagosome lysis (Machado et al., 2014).

Galectin-3 (Gal-3) is a β-galactoside-binding protein with multiple functions including regulation of inflammation, cell growth, signaling, chemotaxis, cell-matrix interactions, tumor progression, and metastasis (Rabinovich et al., 2002; Ochieng et al., 2004). Moreover, authors have observed that *T. cruzi* infection enhances the expression of Gal-3 both *in vivo* and *in vitro* (Acosta-Rodriguez et al., 2004; Vray et al., 2004). Human galectin-3 appears to recognize glycans present in some pathogens and can bind to *T. cruzi* trypomastigote surface proteins (Moody et al., 2000). Recently, authors have proposed that lack of Gal-3 prevents cardiac fibrosis and effective immune responses in *T. cruzi* experimental infection (Pineda et al., 2015). Herein, we provide conflicting results that demonstrated the beneficial impact of Gal-3 expression to the control of infection and to limit heart tissue damage.

## Materials and methods

### Animals

Six- to eight-week-old C57BL/6 wild-type (WT) and Galectin-3 knockout (Gal-3 KO) mice male or female were maintained under standard conditions on a 12-h light-dark cycle in a temperature controlled setting (25°C) with food and water *ad libitum*.

### Ethics statement

Maintenance and care of animals complied with the guidelines of the Laboratory Animal Ethics Committee from the Universidade Federal de Uberlândia. Animal euthanasia was performed based on international welfare grounds according to the American Veterinary Medical Association Guidelines on Euthanasia. This study was approved by the ethics committee for animal research at Universidade Federal de Uberlândia.

### Parasite and cells

*T. cruzi* from the CL strain was maintained in Vero cells (Banco de Células do Rio de Janeiro) cultured in DMEM (Sigma Aldrich) supplemented with 2.5% fetal bovine serum. Peritoneal macrophages from C57BL/6 WT and Gal-3 KO mice were harvested from the peritoneal cavity in 5 mL DMEM, mice were stimulated with 1 mL 3% thioglycollate medium 3 days prior to harvest.

Bone marrow-derived cells from femurs and tibias of C57BL/6 mice were used to generate immortalized macrophage (iMo/B6) cell lines. iMo/B6 cells were transduced with shRNA Lentiviral Particles according to the manufacturers' instructions (Santa Cruz Biotechnology, Inc., Dallas, USA). Control shRNA (sc-108080) and Galectin-3 shRNA (sc-35443) were used. After shRNA transduction cells were analyzed by flow cytometry to check the knocking down expression of Gal-3. Transduction and expression analysis were performed as described by Araújo et al. (2016) WT, control knockdown (CTRL KD) and Gal-3 KD cells were used.

Cells were cultured in DMEM (Sigma) supplemented with 10% FBS, 10 mg/mL streptomycin (Sigma), 100 U/mL penicillin (Sigma), and 40 mg/mL gentamycin (Sigma) at 37°C in a 5% CO_2_ humid atmosphere.

### *In vitro* invasion and multiplication assay

Peritoneal macrophages from WT and Gal-3 KO mice and WT, CTRL KD and Gal-3 KD iMo/B6 were seeded onto 24 wells plate (10^5^ cells/well) containing 13 mm round coverslips and left overnight. After, tissue culture derived trypomastigote forms (TCT; 5 parasites/cell) were placed in contact with cells for 2 h. Wells were washed three times with PBS to remove non-internalized parasites. For invasion analysis (2 h post-infection) and multiplication analysis (24, 48, 72, 96, 150 h post-infection) cells were fixed with Bouin solution, Giemsa stained, and coverslips glued onto glass slides. Number of internalized parasites in a total of 100 cells and multiplication in a total of 100 infected cells were counted. The experiment was performed three times, with triplicates.

### Parasites released

Peritoneal macrophages from WT and Gal-3 KO mice were plated onto 24 wells plate (10^5^ cells/well) and incubated with TCT at parasite/host cell ratio of 5:1, for 2 h. Cells were washed three times with PBS to remove non-internalized parasites. After 96 h the number of parasites released in the extracellular medium was counted in Neubauer chamber.

### Cell viability

Viability of WT and Gal-3 KO peritoneal macrophages infected or not by *T. cruzi* was evaluated using flow cytometry. Cells were seeded at 5 × 10^5^/well in 6-well micro-plates overnight and incubated with TCT (5 parasites/cell) for 2 h when non-internalized parasites were washed with PBS buffer. After 96 h, the cells were washed with PBS, incubated for 30 min with 500 μL of propidium iodide solution (50 μg/mL), washed with PBS, fixed with 4% para-formaldehyde for 1 h and the median fluorescent intensity (MFI) of propidium iodide was determined.

### Leukocytes from the peritoneal cavity

To analyze the recruitment of inflammatory cells and the number of viable parasites in the peritoneal cavity in WT and Gal-3 KO animals, we injected 10^5^ TCT into peritoneal cavity. After 72 h, animals were euthanized by cervical dislocation and peritoneal lavage was performed with 5 ml of cold DMEM supplemented with 10% FBS. Peritoneal leukocytes and free parasites in peritoneal cavity were counted in a Neubauer chamber.

### Animal infection

C57BL/6 WT and Gal-3 KO mice 6–8 weeks of age were infected with TCT as described above. A group of uninfected mice was used as negative control. Animals were euthanized 15 days (acute phase) or 90 days (chronic phase) post infection and spleens and hearts removed. Thereafter, part of each organ was placed in 1 ml of protease inhibitors cocktail composed of AEBSF, Aprotinin, Bestatin, E-64, EDTA, and Leupeptin (cOmplete,Sigma-Aldrich) for the measurement of cytokines by ELISA and the remaining portion used for histological analysis.

### Parasitemia

At 0, 7, 15, 30, 60, and 90 days post-infection blood samples were obtained from each mouse via tail vein to measure parasitemia (amount of parasites in the blood) in WT and Gal-3 KO mice. Approximately 5 μl of blood was mounted on a microscopic slide and total number of parasites counted.

### Cytokine expression

Cytokine levels in tissues from WT and Gal-3 KO control and *T. cruzi* infected mice was performed by macerating spleen and heart then analyzing cytokine levels in the supernatant using ELISA. The organs were kept in 1 ml of protease inhibitor cocktail (1 tablet diluted in 50 ml of PBS—Complete, Sigma Aldrich). Levels of IFN-γ, TNF-α, IL-1β, and IL-4 were measured using ELISA kit from BD Biosciences®.

### Histological analysis

Hearts were processed for histological analysis: organs were fixed in formaldehyde (10% in PBS), then dehydrated in increasing concentrations of ethanol, diaphanized in xylene, and finally embedded in paraffin. Organs were sliced into 5 mm thick sections, placed onto glass slides then stained.

### Toluidine blue staining

To analyze the number of recruited mast cells, sections were stained with toluidine blue. Briefly, xylene deparaffinized sections were rehydrated by a rinse in 96% ethanol, 10 min in 70% ethanol and 3 rinses in ultrapure water. Fully hydrated sections were then placed in phosphate citrate buffer pH 3.0 for 5 min and stained with 0.5% toluidine blue for 3 min. Excess dye was removed by dipping the slides in buffer and samples were clarified by increasing of ethanol and xylene concentrations and slides were mounted with Entellan®. Data were analyzed using the ratio of the total number of mast cells per area of histological section (total number of mast cells/cm^2^).

### Picrossirius staining

To examine the role of Gal-3 in fibrosis, collagen was stained with picrosirius. Sections were subjected to successive immersions in xylene, hydrated in decreasing ethanol, and water concentrations and stained with picrossirirus solution (Sigma Aldrich) for 50 min and washed in distilled water. Next, sections were placed in hematoxylin solution for 4 min, washed in water, and stained for 1 min with aqueous eosin. After dehydration in ethanol xylene diaphanization, slides were mounted with Entellan®. Quantification of total collagen fibers was performed using Image J software; images of 30 fields of each sample were taken with a light microscope (Nikon). According to the literature collagen stained in red under polarizing microscope indicates the type I and in green type III. In this way, red and green channels of each image acquired under polarized light to infer the two types of collagen in the tissue sections were quantified. For this analysis 20 fields were quantified for each sample.

### RNA extraction, reverse transcription, and real-time RT-PCR

Total RNA was isolated from mice hearts using the RiboZol® Plus RNA Purification Kit (Amresco) according to the manufacturer's instructions. RNA was eluted in 20 μl of elution buffer and stored at −80°C until further use. For complementary DNA (cDNA) synthesis we used High Capacity cDNA Reverse Transcription Kit (Applied Biosystems) according to the manufacturer's recommendations.

Gene expression of TGF-β, collagens type I and III was performed using the SYBR® Green PCR Master Mix (2X) (Applied Biosystems), 10 μM forward and reverse primer (0.5 μL F + 0.5 μL R), 4 μL nuclease-free water and 2 μL cDNA (125 ng/μL). For quantitative RT-PCR was used ABI 7300 equipment (Applied Biosystems) and SDS v1.4.1 Software (Applied Biosystems) to analyze received data. Data were normalized using GAPDH as a housekeeping gene and then analyzed by comparative threshold cycle (CT) method to calculate fold changes of expression in infected groups compared with control group (relative quantity, 2^−ΔΔCT^). Primers sequences were designed using sequence alignments obtained at NIH/NCBI gene bank based in the RNA published sequences.

### Statistical analysis

*In vitro* experiments were performed at least three times in triplicate. *In vivo* experiments were carried out twice using five animals per group. Samples from each animal were individually analyzed twice in triplicate. All data were first checked for normal distribution. Significance differences were determined by Kruskal–Wallis and Dunn's multiple comparisons test (GraphPad Prism Software version 6.01). Data were considered statistically significant at *p* < 0.05.

## Results and discussion

### Lack of Gal-3 expression enhances parasite intracellular replication

**Figure 1:**
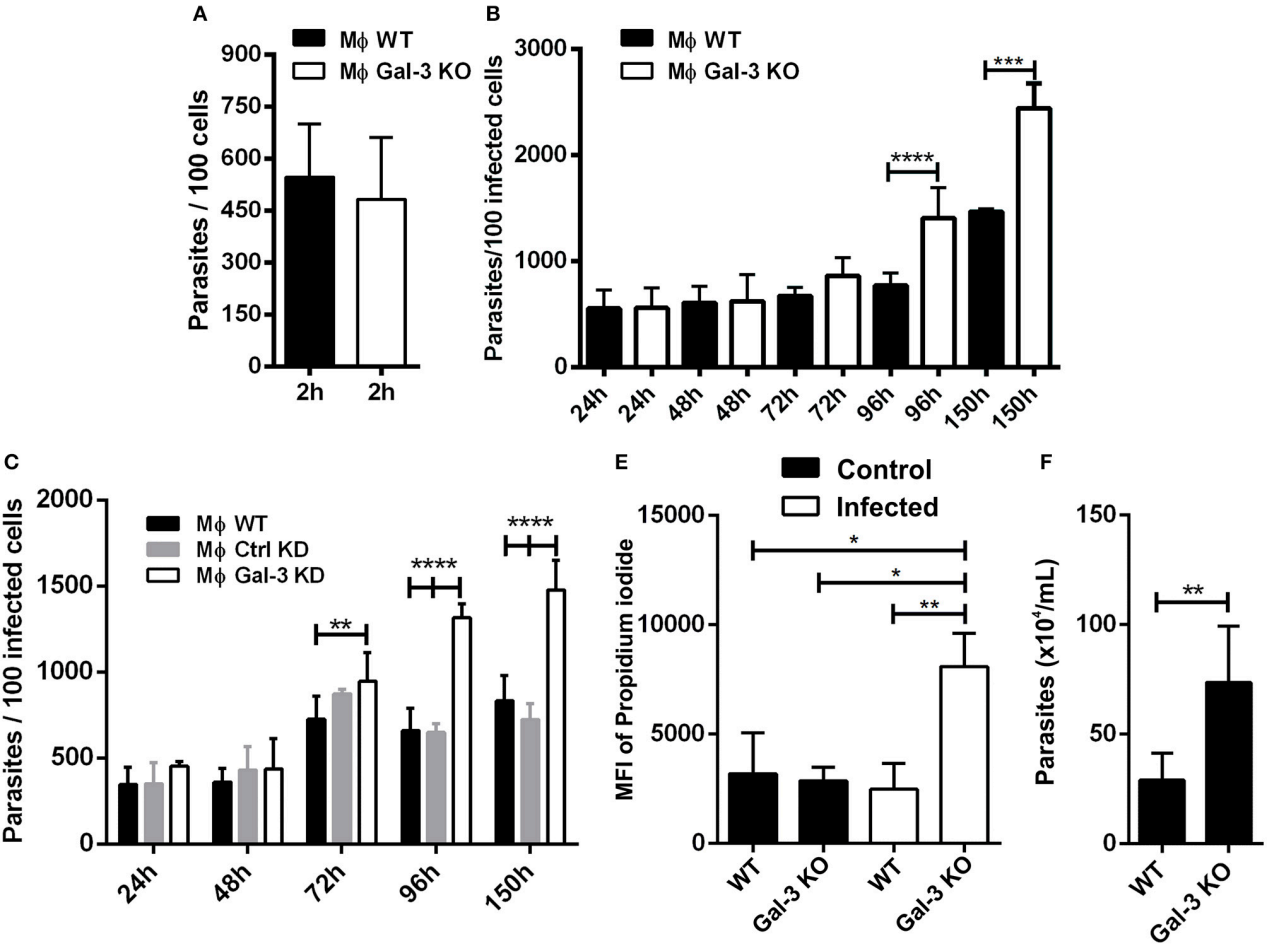
Lack of Gal-3 increased parasite replication and host cell lyses. Mouse peritoneal macrophages were plated and submitted to invasion. At 2 h after invasion and additional later time points, coverslips were washed, fixed with Bouin, and stained with Giemsa. For invasion analysis after 2 h of infection was counted the number of parasites in 100 infected peritoneal macrophages **(A)**. The number of parasites in 100 peritoneal macrophages **(B)** and in 100 iMo/B6 cells **(C)** was counted after 24, 48, 72, 96, and 150 h of infection. The percentage of lysed cells was determined by the nuclear incorporation of propidium iodide by flow cytometry **(D)**. The number of parasite released in the supernatant of infected macrophages at 96 h post-infection was counted **(E)**. Comparisons between WT and Gal-3 KO were performed by using Kruskal–Wallis and Dunn's multiple comparisons test. ^*^p < 0.05, ^**^p < 0.01, ^***^p < 0.001, ^****^p < 0.0001.

Researchers have observed that Gal-3 KO macrophages are deficient in the phagocytosis of IgG-opsonized erythrocytes and apoptotic cells during the initial time points of interaction. However, after 1 h of macrophage and particle interaction, galectin-3-deficient phagocytes exhibited WT phagocytosis ability. In addition, Gal-3 KO peritoneal macrophages displayed attenuated phagocytic clearance of apoptotic thymocytes *in vivo* (Sano and Liu, 2001). In agreement with this study, we verified that after 2 h of interaction between peritoneal macrophages with *T. cruzi*, both WT and Gal-3 KO displayed similar phagocytosis indexes compared to control groups (Figure 1A).

One intriguing observation in our *in vitro* studies was that at later time points, *T. cruzi* showed a higher proliferation index in Gal-3 KO macrophages and iMo/B6 Gal-3 KD (Figures 1B,C). In this sense, Gal-3 expression may contribute to impair parasite intracellular multiplication. Accordingly, in its absence host cells would be lysed to a higher extent. In order to confirm this hypothesis we measured the number of parasites released in the supernatant of infected macrophages and also cell viability. Our results showed a higher percentage of lysed cells from Gal-3 KO mice (Figure 1D), coinciding with an enhanced release of parasites (Figure 1E). Thus, we provided *in vitro* the first evidence that supports the notion that Gal-3 plays a pivotal role in controlling *T. cruzi* proliferation and limiting host cell lyses.

### Gal-3 KO mice infected with *T. cruzi* showed higher systemic parasitemia and delayed clearance of intraperitoneal parasites

**Figure 2:**
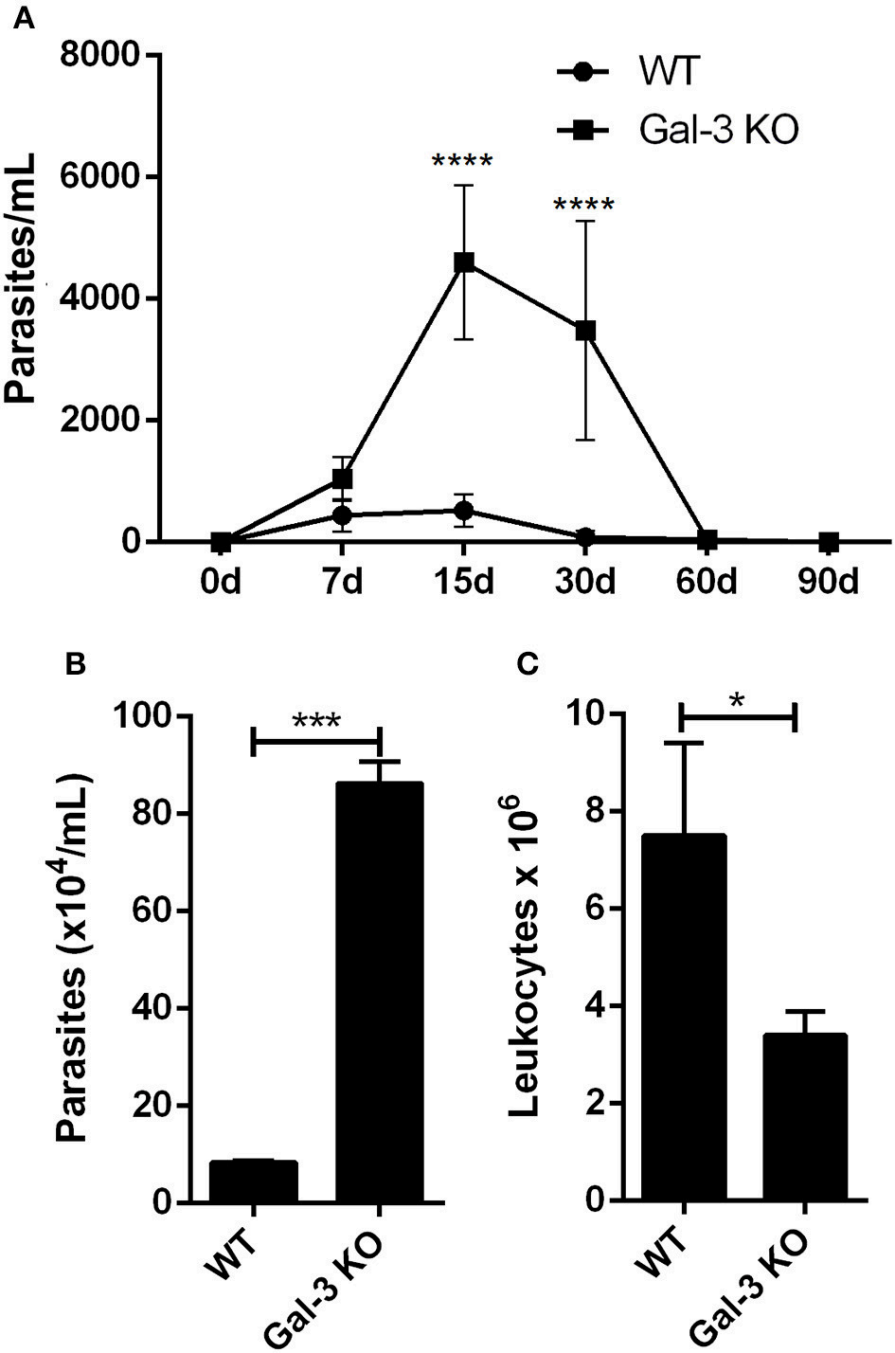
Lack of Gal-3 leads to higher systemic parasitemia, delayed parasite clearance, and low leukocyte recruitment to the peritoneal cavity. Mice were inoculated intraperitoneally with *T. cruzi* CL strain trypomastigotes. At different time points post-inoculation parasitemia was measured in tail peripheral blood samples **(A)**. Parasites released **(B)** and leukocyte recruitment **(C)** to the peritoneal cavity was counted after 96 h of infection. **(A)** Comparisons between groups were performed by Kruskal–Wallis and Dunn's multiple comparisons test. ^*^p < 0.05, ^***^p < 0.001, ^****^p < 0.0001.

Corroborating our *in vitro* results, Gal-3 KO mice displayed higher levels of parasitemia than WT animals (Figure 2A). This phenotype may result from the higher parasite proliferation rate within Gal-3 KO cells and/or by an inappropriate host immune response by Gal-3 KO mice during the acute phase of the infection. It is important to note that by 30 days post-inoculation, while WT animals had controlled parasitemia, Gal-3 KO mice still displayed high number of parasites in the bloodstream (Figure 2A) indicating that Gal-3 expression favors the control of parasite burden in *in vivo T. cruzi* infection. Similar results were observed for galectin-1 (Gal-1), another member of the galectin family, which expression reduced infection by *T. cruzi* (Benatar et al., 2015). Authors found that Gal-1 levels were higher in sera from patients with chronic Chagas' disease compared with non-infected subjects but no differences were observed between cardiac patients and asymptomatic individuals. In agreement with our results they demonstrated by *in vivo* experiments that mice deficient in Gal-1 and inoculated with *T. cruzi* infective forms showed higher parasitemia and lower survival rates than WT animals. Then, these data reinforce the importance of some lectins in controlling *T. cruzi* infection.

Conversely, authors have shown that Gal-3 was upregulated during experimental infection with *T. cruzi* from Y strain. Moreover, they observed that Gal-3 KO mice showed an increased number of circulating parasites at day 10 after infection, but eliminated parasites at later stages similarly to WT controls. It was hypothesized that the increased number of parasites in the bloodstream of Gal-3 KO animals could be accounted for by defective early parasite recognition by the innate immune system (Pineda et al., 2015).

In order to get additional insights about the impact of Gal-3 on host immune response activation against *T. cruzi*, we first evaluated the effect of Gal-3 expression on intraperitoneal macrophage recruitment and parasite uptake. For this purpose, we collected peritoneal content 3 days after infection, counted the number of viable parasites and total leukocytes. In agreement with Sano and Liu (2001), we noted a delayed clearance of parasites (Figure 2B) and a lower recruitment of leukocytes into the peritoneal cavity (Figure 2C) in Gal-3 KO animals as compared to WT mice.

However, when we measured IL-1β, TNF-α, IFN-γ, and IL-4 expression levels in spleen and heart samples from infected WT and Gal-3 KO mice during both acute and chronic phases of *T. cruzi* infection, we observed that IL-1β (Supplementary Figure 1A) and IFN-γ (Supplementary Figure 1E) were upregulated only in spleen from wild-type mice during acute infection. The levels of TNF-α (Supplementary Figure 1C) and IL-4 (Supplementary Figure 1G) were not altered in the spleen in acute infection. In heart samples we observed increased secretion of TNF-α (Supplementary Figure 1D) and IFN-γ (Supplementary Figure 1F) in WT animals while in Gal-3 KO mice we detected an increased IL-1β (Supplementary Figure 1B) and IL-4 (Supplementary Figure 1H) production. These results suggested that wild type animals could trigger a predominant Th-1 acquired immune response. This phenotype may have favored the parasitemia clearance by the 30 day post-infection. Contrariwise, the high levels of IL-4 expression in the heart from Gal-3 KO mice during acute infection may have accounted for the reduction of Th1 immune response. Thus, this phenotype is consistent with animals more susceptible to infection and supports the high and prolonged blood stream parasitemia.

During the chronic phase of infection we observed no significant variations in IL-1β, TNF-α, IFN-γ, or IL-4 concentrations in spleen regardless of Gal-3 expression (Supplementary Figures 2A,C,E,G). We also observed that heart tissue of infected WT mice showed a Th2-skewed response with basal levels of TNF-α (Supplementary Figure 2D) and IFN-γ (Supplementary Figure 2F), decreased levels of IL-1β (Supplementary Figure 2B), and increased IL-4 levels (Supplementary Figure 2H). This profile observed in WT animals can be considered an appropriate immune regulation mechanism that may account to low heart damage. Our results are supported by previous studies which showed that galectin-3 exerts a cytokine-like regulatory activity (Jeon et al., 2010). Also, Gal-3 KO mice exhibited impaired neutrophil recruitment and diminished macrophage survival (Colnot et al., 1998). It has also been reported that Gal-3–deficiency promotes a reduced NF-kB response and decreased cytokine production (Hsu et al., 2000; Chen et al., 2006). Pineda et al. (2015) also observed that Gal-3 KO animals displayed defects in Th1 and Th2 immune response. An intriguing question may be raised: what would be the mechanism triggered by the Gal-3 KO mice to control parasite burden and tissue damage? On the contrary, we have stressed the important role of galectin-3 in controlling parasite burden and in modulating Th1/Th2 host immune response.

### Gal-3 KO mice infected with *T. cruzi* showed higher heart fibrosis

**Figure 3:**
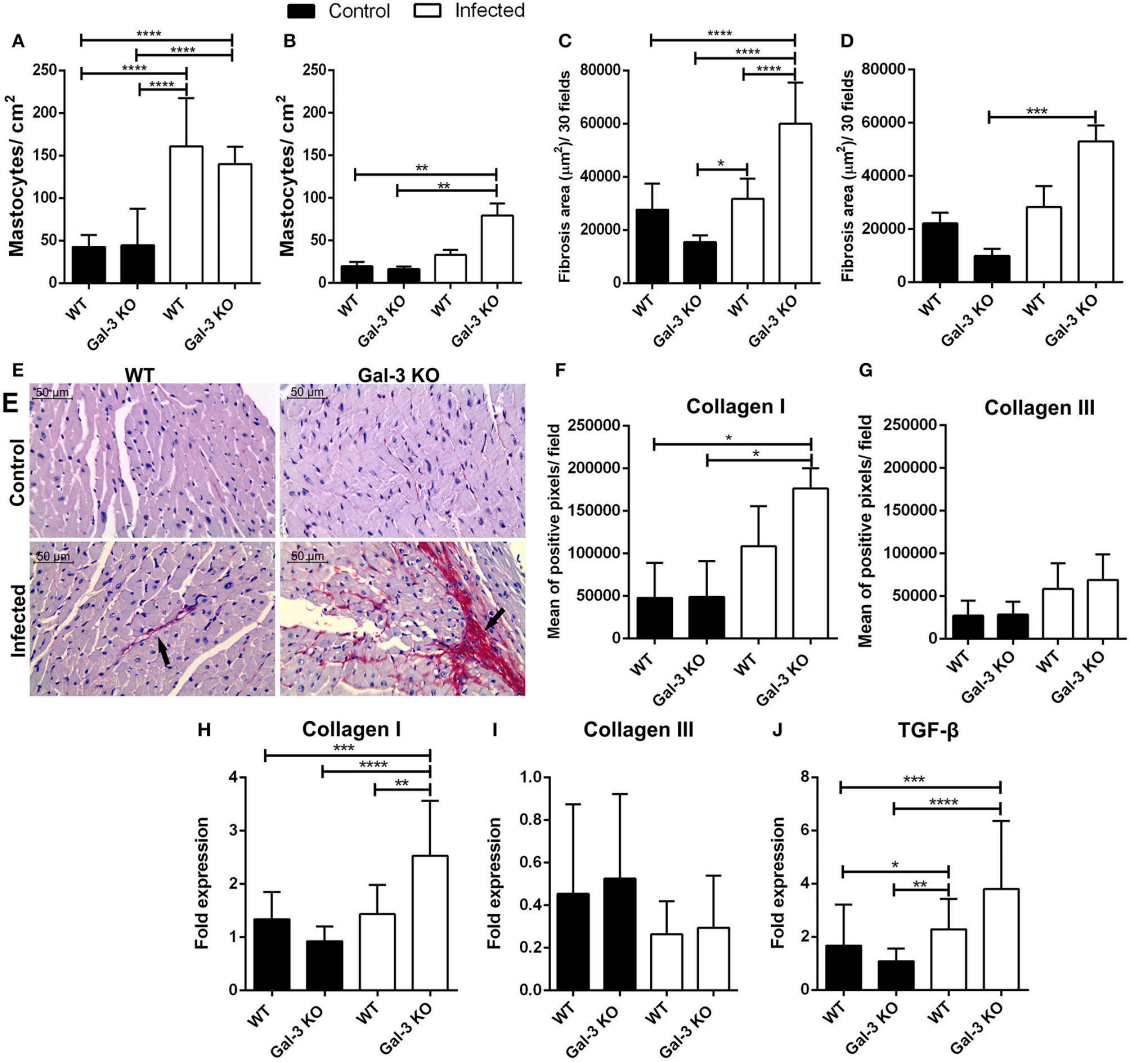
Lack of Gal-3 leads to recruitment of mast cells and higher heart fibrosis levels during the chronic stage of *T. cruzi* infection. Cardiac tissue sections from WT or Gal-3 KO mice infected or not by *T. cruzi* CL strain trypomastigotes were stained with toluidine blue for mast cell count or picrosirius for quantification of collagen. Mastocytes recruitment during acute (15 days post infection) **(A)** and chronic (90 days post infection) **(B)** infection was count and infected Gal-3 KO mice presented higher recruitment in both phases. Augmented/increased levels of fibrosis were observed in heart sections from these animals during acute **(C)** and chronic **(D)** phases of infection. Representative histological images from heart of WT and Gal-3 KO mice infected or not by *T. cruzi* for 90 days **(E)**. Red staining represents fibrosis (black arrows). Sections stained with picrosirius were analyzed under a polarized microscope for differentiation of collagen type I **(F)** and III **(G)**. Collagen I **(H)**, collagen III **(I)**, and TGF-β **(J)** RNA levels were quantified by Real-Time RT-PCR in hearts. Chronically infected mice show higher amounts of collagen type I in heart, whereas Gal-3 KO animals have higher levels compared with WT. The infection did not alter the levels of collagen type III in heart. TGF-β gene expression was increased in Gal-3 KO mice. Comparisons between groups were performed by Kruskal–Wallis and Dunn's multiple comparisons test. ^*^p < 0.05, ^**^p < 0.01, ^***^p < 0.001, ^****^p < 0.0001.

**Figure 4:**
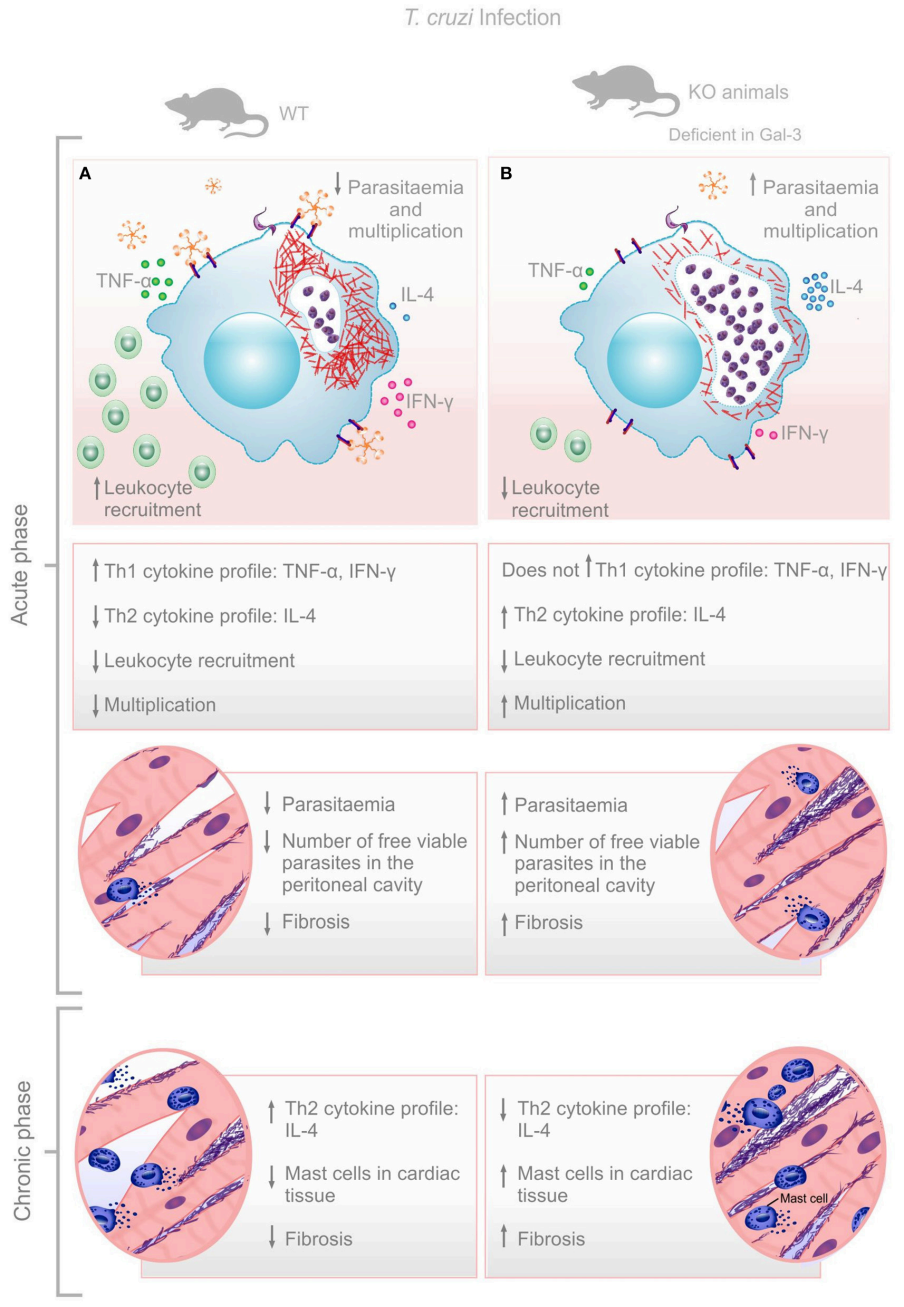
Envisaged mechanism involving Gal-3 in *T. cruzi* infection control. Gal-3 expression ensures macrophage recruitment, parasite clearance, and balanced Th1/Th2 immune response leading to low bloodstream parasitemia and rapid control of infection with low cardiac damage and fibrosis **(A)**. Lack of Gal-3 expression lead to low macrophage recruitment, low parasite clearance, and unbalanced Th1/Th2 immune response leading to high bloodstream parasitemia and delay control of infection with high cardiac damage and fibrosis **(B)**.

Finally, we addressed the impact of these different phenotypes in heart tissue damage. For that purpose, we determined the mast cell infiltrate and fibrosis area in heart samples from WT and Gal-3 KO mice. We observed increased recruitment of mast cells in WT and Gal-3 KO animals during the acute phase of infection (Figure 3A) but in chronic phase only Gal-3 KO animals showed elevated levels of mast cells in the heart (Figure 3B). In accordance to studies that have shown an important role of mast cells to *T. cruzi* pathology (Meuser-Batista et al., 2008). Of note, though the levels of the control groups (WT and Gal-3 KO mice) are lower in the chronic phase of infection (Figure 3B) compared to the acute phase (Figure 3A), it seems that the mast cell recruitment decreased in WT mice (3-fold increase in Figure 3A and 1.5-fold in Figure 3B compared to control mice) and remained high/increased in Gal-3 KO mice (3-fold increase in Figure 3A and 4-fold in Figure 3B compared to control mice).

Interestingly, it was previous reported that mast cells release tryptase and thrombin that enhance the differentiation of human fibrocytes and initiate the formation of collagen fibers in damaged tissue (White et al., 2015). This information prompted us to accurately verify fibrosis in heart tissue from our experimentally infected animals. Thus, we analyzed collagen content in histological specimens and observed that during both acute and chronic phases, there are larger areas of fibrosis in infected Gal-3 KO animals if compared to WT mice (Figures 3C–E). Collagen fibers in knockout animals chronically infected were mainly composed of collagen type I (Figures 3F,G) without alteration of type III (Figures 3H,I).

In addition, we evaluated the gene expression of Transforming Growth Factor ß (TGF-ß). We have seen that in the chronic phase there is an increase in TGF-β in the heart of Gal-3 KO mice when compared to control group (Figure 3J). This cytokine is associated with increased collagen deposition (Dutra et al., 2009). Thus, corroborating its upregulation in Gal-3 KO infected mice. In this sense, the expression of TGF-β in areas close to tissue injuries may contribute to the deposition of extracellular matrix components in order to replaced parasite lysed cells and cardiac muscular fibers.

Pineda et al. (2015) provided the following hypothesis to explain higher heart tissue fibrosis in WT infected mice: “Hence, Gal-3 may trigger self-destructive mechanisms to initiate cardiac inflammation, and perhaps subsequent CCC.” However, their study focused in two time points of acute infection. They did not extended to chronic infection such as ours that performed all experiments in animals at the 15th day and the third month post-infection. This might be the major factor for these contradictory results. Conversely, we have used a higher number of parasites for the *in vivo* experimental infections. In this sense, higher levels of parasites could also contribute to the enhanced fibrosis in Gal3 KO mice, given that extracellular vesicles released by *T.cruzi* can activate both the innate and adaptive compartment, whereby they are found to be very pro-inflammatory in the chronic phase (Nogueira et al., 2015). This in turn might explain higher pathogenicity in the Gal3 KO mice observed herein.

It is worth mentioning that significant heart damage occur mainly along the chronic phase of the infection. Moreover, Noya-Rabelo et al. (2017) analyzed the clinical correlation between plasma levels of Gal-3 and myocardial fibrosis, and concluded that despite fibrosis varied within different clinical forms of Chagas' disease, the median Gal-3 concentration was similar in infected groups. Thus, *T. cruzi* infection induces an increase in Gal-3 expression, but this increase is not directly related to fibrosis.

Although, the impact of Gal-3 during *T. cruzi* infection is still puzzling, here we verified that this lectin promotes leukocyte recruitment, parasite replication control and balanced Th1/Th2 immune response resulting in low cardiac tissue damage at later time points after infection. Gal-3 KO animals had impaired leukocyte recruitment to the peritoneal cavity, higher bloodstream parasitemia during acute phase, and increased recruitment of mast cells and fibrosis of the cardiac tissue during the chronic phase of the infection. Higher parasite replication in Gal-3 KO animals may lead to increased parasite release from lysed infected cells which is replaced by the collagen deposition (Figure 4). In this sense, we do believe that effects shown in the chronic phase are knock-on effects from the events observed during the acute phase which demonstrated the important role of Gal-3 in controlling parasite burden and limiting tissue damage.

## Author contributions

AAS, TLT, SCT, FCM, MAS, PCBT, RTSB, FAM, NSLS, and CCR: Performed experiments, formatted data sets, and analyzed data. TCT: Gave access to the histological facility and helped in samples processing and data analysis. AAS, MCRB, RAM, DSL, and VMRA: Draft the manuscript, interpreted data, and contributed to the intellectual content of the manuscript. CVS: Coordinated the project, wrote the manuscript, interpreted data, and got specific grants for the project development. FAM, NSLS, CCR: Prepared reagent solutions and performed experiments.

### Conflict of interest statement

The authors declare that the research was conducted in the absence of any commercial or financial relationships that could be construed as a potential conflict of interest.

## Supplementary material

Supplementary Figure 1 Pro-inflammatory cytokine expression was down-regulated in spleen and heart tissue of infected Gal-3 KO mice during the acute phase of infection. IL-1β **(A,B)**, TNFα **(C,D)**, IFNγ **(E,F)**, and IL-4 **(G,H)** from the supernatant of macerated spleen and heart was measured using ELISA. Comparisons between groups were performed by using Kruskal–Wallis and Dunn's multiple comparisons test.^*^*p* < 0.05, ^**^*p* < 0.01, ^***^*p* < 0.001, ^****^*p* < 0.0001.

Supplementary Figure 2 Pro-inflammatory cytokine expression profiles were observed in spleen and hearts of either WT or Gal-3 KO infected animals during chronic phase of infection. IL-1β **(A,B)**, TNFα **(C,D)**, IFNγ **(E,F)** and IL-4 **(G,H)** were measured by ELISA from the supernatant of macerated spleen and heart. Comparisons between groups were performed by using Kruskal–Wallis and Dunn's multiple comparisons test. ^*^*p* < 0.05, ^**^*p* < 0.01, ^***^*p* < 0.001, ^****^*p* < 0.0001.
