# Supplementary figures and images for: Galectin-3: A Friend but Not a Foe during Trypanosoma cruzi Experimental Infection
Source: Front Cell Infect Microbiol. 2017 Nov 3;7:463. doi: 10.3389/fcimb.2017.00463 (PMC5675870; doi:10.3389/fcimb.2017.00463)

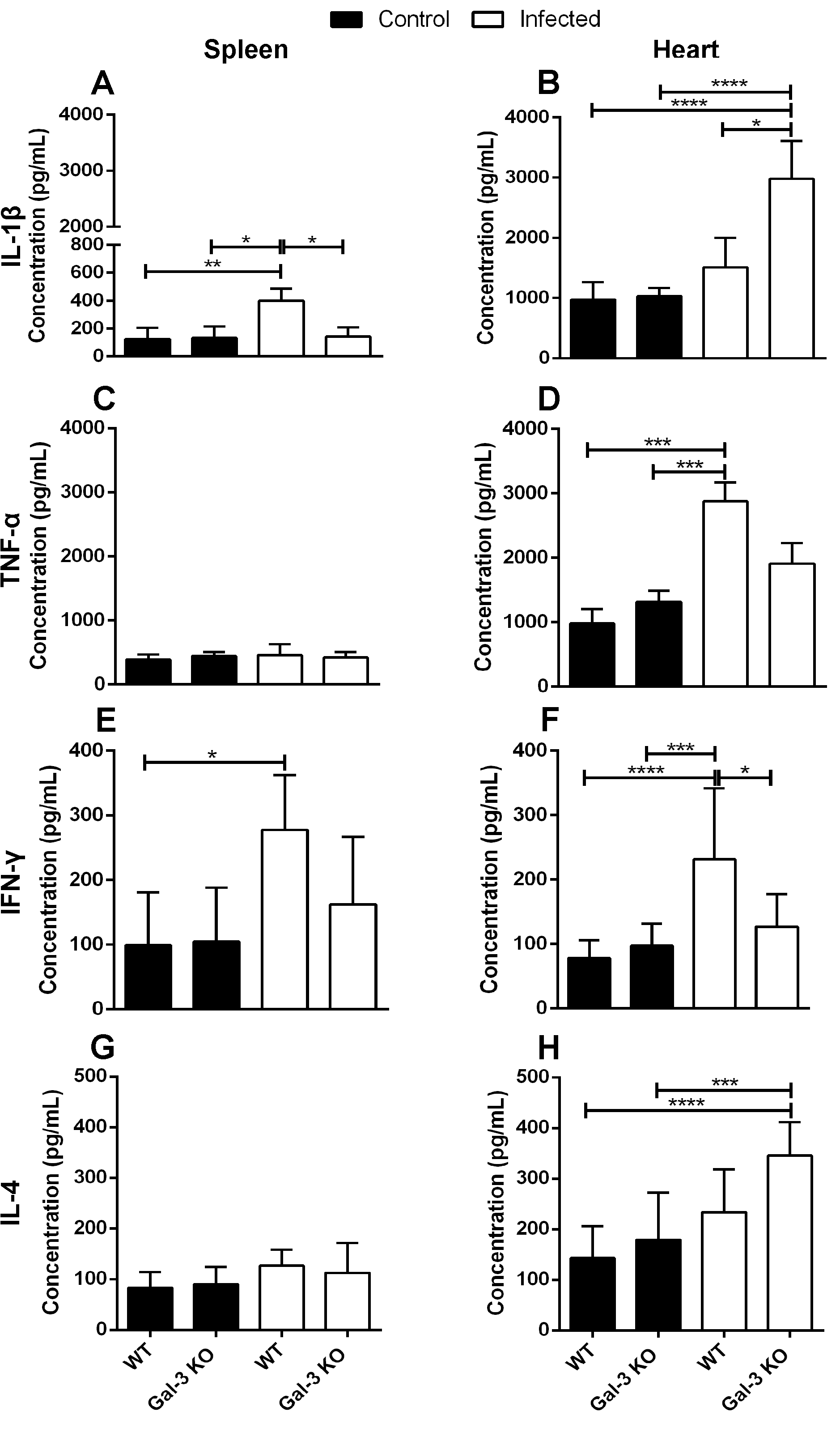

Supplement: Supplementary Figure 1 — Pro-inflammatory cytokine expression was down-regulated in spleen and heart tissue of infected Gal-3 KO mice during the acute phase of infection. IL-1β (A,B), TNFα (C,D), IFNγ (E,F), and IL-4 (G,H) from the supernatant of macerated spleen and heart was measured using ELISA. Comparisons between groups were performed by using Kruskal–Wallis and Dunn's multiple comparisons test.*p < 0.05, **p < 0.01, ***p < 0.001, ****p < 0.0001. [file Image1.TIF]

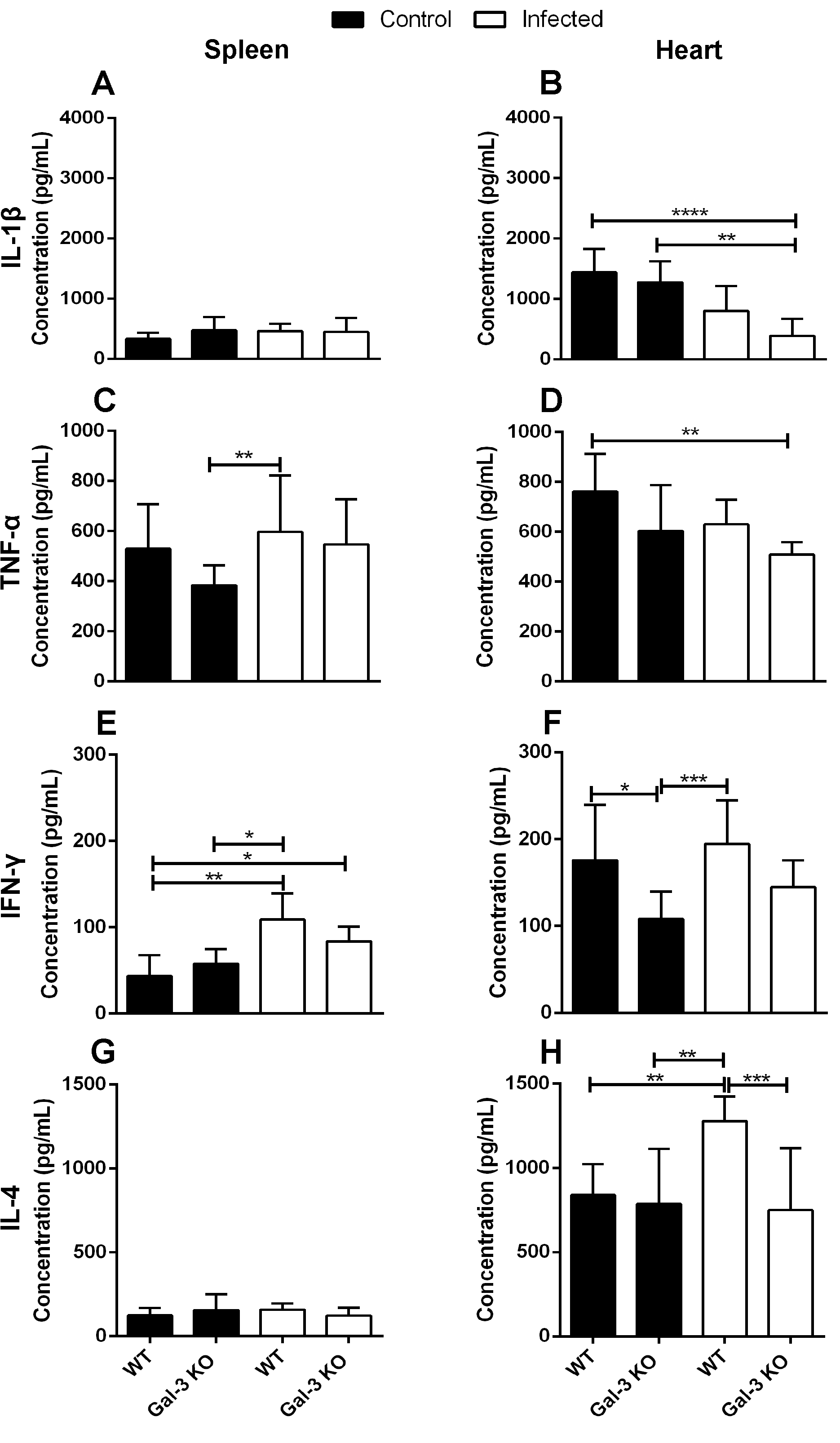

Supplement: Supplementary Figure 2 — Pro-inflammatory cytokine expression profiles were observed in spleen and hearts of either WT or Gal-3 KO infected animals during chronic phase of infection. IL-1β (A,B), TNFα (C,D), IFNγ (E,F) and IL-4 (G,H) were measured by ELISA from the supernatant of macerated spleen and heart. Comparisons between groups were performed by using Kruskal–Wallis and Dunn's multiple comparisons test. *p < 0.05, **p < 0.01, ***p < 0.001, ****p < 0.0001. [file Image2.TIF]
